# Tetanus Caused by the Administration of a Powerful Dose of Arnica

**Published:** 1854-03

**Authors:** Leopold Turck


*Tetanus caused by the administration of a powerful dose of arnica.* By Dr. Leopold Turck.—Indigneous medicinal plants to which our attention has been called in your excellent journal, would receive much more of our favor and be much more useful were they gathered with more judgment and care. Those which merely furnish leaves and roots are picked indiscriminately in spring, summer or autumn, though experiences teaches us that the qualities of plants vary remarkably according to the different stages of their vegetation; nor is any regard paid to the country whence these plants are derived, though climate is known to exercise the greatest influence upon the quality of their roots, flowers and plants. This influence is so great indeed, that the same plant may become edible in one, which was poisonous in another; an highly aromatic plant from the South, be but slightly fragrant in the North; but to return to our subject.

Meline (Louis) des Granges de Plombieres, aged 28 years, large and powerful, and of excellent health, carried, on the 21st July, 1849, so heavy a load, as to occasion pains in the back and oppression, unaccompanied, however, with spitting of blood. His mother gave him to drink a strong infusion of arnica. It appears that she had put a large handful of the flowers, recently gathered, of this plant, into a pint of boiling water. Soon afterwards Meline experienced a general agitation, which increased to such an extent, that the fourth day a general tetanus set in. His wife and he attributed all these syptoms to the strong infusion he had drunk seven days previously. By the aid of inhalation of chloroform, I caused the tetanus to cease momentarily, and my patient from this time experienced no further suffering. The symptoms disappeared the first time of its exhibition, for two hours; the second time, for an hour only; the last time, for hardly half an hour. Meline died on the 1st of August. He was not opened. There was no external indication of any injury; if he had any, very certainly the violent action of the arnica had exerted upon it a baneful influence, such, in short, as the patient himself attributed to it.

I have known arnica, taken in large doses by a young girl for improper purposes, produce very violent abdnominal pains, simulating peritonitis, and complicated with a general nervous agitation. In this instance I was enabled to save both mother and child.

Dr. Grillott, one of our colleagues, has seen vertigo so severe, for several hours, as to prevent the patient from either sitting or standing, produced by an over-dose of arnica.—*Revue de Therapeutique.*

